# Presence and possible significance of immunohistochemically demonstrable prolactin in breast apocrine metaplasia.

**DOI:** 10.1038/bjc.1987.60

**Published:** 1987-03

**Authors:** S. Kumar, R. E. Mansel, B. Jasani

## Abstract

**Images:**


*Br. J. Cancer* (1987), **55**, 307–309 © The Macmillan Press Ltd., 1987

### Presence and possible significance of immunohistochemically demonstrable prolactin in breast apocrine metaplasia

S. Kumar[1]*, R.E. Mansel[1] & B. Jasani[2]

*Departments of [1]Surgery and [2]Pathology, University of Wales College of Medicine, Cardiff CF4 4XN, UK.*

**Summary** Paraffin wax embedded formalin-fixed benign breast disease tissue taken from 17 patients (15 with microcystic disease and 2 with fibroadenoma) was studied for the presence of tissue bound prolactin using a rabbit antiserum against human prolactin applied in conjunction with a highly sensitive modified version of the dinitrophenyl (DNP)-hapten sandwich staining (DHSS) procedure. Sections taken from 14 of the 15 cases showing apocrine cystic changes exhibited strong prolactin staining restricted to the cytoplasm of metaplastic apocrine cells lining the cysts. Normal lobules and ducts and blunt duct proliferations were all negative, as were also the two cases of fibroadenoma. In contrast 6 out of 8 cases of breast cancer examined showed heterogenously distributed cytoplasmic staining in the cancer cells.

Maximal prolactin staining in the apocrine cells was observed at antiserum dilutions as high as 1:60,000. This compared favourably with a 1:120,000 dilution that gave maximal levels of staining in the prolactotrophs present in serial sections taken from formalin fixed paraffin wax embedded post mortem human anterior pituitaries. In both types of tissues the specific staining was abolished by pre-absorption of the antiserum with human prolactin ($10\,\mu g\,ml^{-1}$). No staining was observed when the anti-prolactin serum was either omitted or substituted with DNP-labelled normal rabbit serum.

Apocrine metaplasia in cystic disease of the breast has recently been found to be associated with an increased breast cancer risk. The strong and selective presence of immunohistochemically demonstrable prolactin in the metaplastic cells may be of significance in view of the hormone's known growth stimulating effect on the breast epithelium.

For a long time apocrine metaplasia (or pink cell change), associated with cystic breast epithelium, has been regarded as having little or no significance in relation to malignant breast disease. However, recent reports from 4 independent groups suggest that apocrine cell metaplasia, in particular papillary apocrine change, may represent a minor but pathogenetically important marker of breast cancer risk (Page *et al.*, 1978; Roberts *et al.*, 1984; Dixon *et al.*, 1985; Page & Dupont 1986; Haagensen 1986).

In normal breast tissue apocrine change is associated with prolactin (PRL) directed milk production (Shiu & Friesen, 1980). In patients with cyclical mastalgia and nodularity a consistent elevated pituitary PRL secretory activity in response to domperidone and TRH provocation tests has been found (Kumar *et al.*, 1984), suggestive of a functional role of PRL in the induction of painful nodularity. Furthermore, in benign breast disease (BBD) subjected to biopsy, nodularity is often associated with microcystic change that shows variable degrees of apocrine metaplastic change in the epithelium lining the cysts (Craigmyle, 1984). The highly focal nature of such a change has prevented direct biochemical examination of a possible role played by PRL. On the other hand, successful identification of PRL presence has recently been reported in breast tissue sections using an immunohistochemical approach (Dhadly & Walker 1983; Purnell *et al.*, 1982; Marchetti *et al.*, 1984). This study was designed to examine whether or not apocrine metaplastic change in BBD is associated with the presence of immunohistochemically detectable PRL.

### Materials and methods

Formalin fixed paraffin wax embedded tissues from 17 selected cases of benign breast disease biopsy were examined. There were 15 cases with microcystic disease and 2 fibroadenomas. Cysts lined by flattened epithelium and varying heights of apocrine metaplastic cells were present in all the 15 cases chosen. Several of the cysts had both types of lining in the same cyst. Although none of the first set of sections showed papillary apocrine change or atypical hyperplasia, examination of a further series of sections (up to 4 in each case) taken from separate blocks showed papillary apocrine change to be present in all the 15 cases with microcystic disease, as defined by the criterion of Page *et al.* (1978). In 2 cases there was evidence of epitheliosis without atypia, 2 had sclerosing adenosis and 8 showed typical blunt duct proliferation. In addition sections from 8 breast carcinoma cases were examined and at least two of these had apocrine change in the background breast tissue. All the cancers were invasive ductal carcinomas. From 8 to 10 semi-adjacent $5\,\mu m$ sections were cut from the paraffin wax blocks and mounted on chrome gel coated glass slides. A section from a formalin fixed paraffin wax embedded post-mortem anterior pituitary was included in each batch as a positive control. Similarly, lymph node and thyroid sections were included as negative controls. The biopsy specimens were from 2 to 8 years old.

### *Immunoperoxidase labelling*

The dinitrophenyl (DNP)-hapten sandwich staining (DHSS) procedure (Jasani *et al.*, 1981) in its modified form (Jasani & Williams, 1985; Jasani *et al.*, 1985) was used throughout for immunoperoxidase labelling of the tissue sections.

Rabbit anti-human prolactin (Rb aPRL; NIAMDD No. AFP C11580) was obtained as a gift from the National Institute of Arthritis, Metabolic and Digestive Disease (Bethesda, Maryland, USA). This was non-deleteriously DNP-labelled as described previously (Hewlins *et al.*, 1984). The resulting DNP a-PRL was tested over a dilution range of 1:15,000 to 1:480,000 using an incubation period of 16 h at 4°C. The secondary detection reagents, IgM anti-DNP monoclonal bridge antibody, DNP-labelled peroxidase conjugate and DNP-glucose oxidase, were each applied for 45 min with washing in phosphate-buffered saline (PBS) in between each step. The colour reaction was developed using diaminobenzidine and the nascent hydrogen peroxide

*Present address: Department of Surgery, King Georges Medical College, Lucknow, India.
Correspondence: B. Jasani.
Received 11th June 1986; and in revised form 28th October 1986.

generated by glucose (Jasani & Williams, 1985; Jasani *et al.*, 1985). All the sections were finally counterstained with Mayer's haemalum, cleared through xylene and mounted permanently in Terpene.

Control studies included omission of DNP-aPRL and substitution with the appropriate dilution of dinitrophenylated normal rabbit serum (DNP-NRS) and incubation with DNP-aPRL preabsorbed with highly pure human PRL $10\,\mu g\,ml^{-1}$ (NIAMDD, AFP 2284C2). Studies were also performed to examine the effect of exposing sections to the highly pure human PRL prior to treatment with DNP-aPRL antibody.

All the slides were examined and assessed for the immuno peroxidase staining independently by two observers (SK and BJ).

### Results

Of the 17 BBD tissue blocks studied, one specimen with only mild cystic changes and two fibroadenomas were negative for specific staining over the DNP-aPRL concentration range examined. The remaining 14 showed the following typical staining characteristics agreed upon by both the observers. Papillary apocrine change was consistently associated with the strongest intensity of staining (Figure 1a). Tall apocrine metaplastic cells lining the cyst walls were also consistently stained well above the background level and the reaction was predominantly localised within the cell cytoplasm. Flattened cyst epithelium showed specific staining in only 2 specimens, the staining being considerably weaker than that associated with the taller cells. Blunt ducts lined by the normal double layer of cells did not stain. Other metaplastic areas, epitheliosis (in 2 specimens) and adenosis (in 2 specimens) stained rather weakly and heterogenously. Morphologically normal lobules and ducts gave no staining. Background staining of the breast parenchyma and collagen was not prominent in the majority of the cases though weak non-specific staining at the periphery of the sections was a commonly observed phenomenon and was ruled out as an edge or a drying artefact.

The optimal staining reaction was obtained at the dilution of 1:60,000 of DNP-aPRL diluted in BSA. A similar level of staining reaction in the anterior pituitary sections was observed consistently at 1:120,000 dilution. Sections of lymph node and thyroid tissue with oxyphil change did not immunostain at any of the dilutions tested. Prior incubation of the sections with human PRL resulted in a markedly enhanced staining level (Figure 1b) similar in intensity to that seen with a higher concentration (i.e. 1:15,000 dilution) of DNP-aPRL serum but without a parallel increase in the background level, thus indicative of a genuine and specific enhancement. In contrast, omission of DNP-aPRL or its substitution with DNP-NRS abolished specific staining as did prior absorption of the DNP-aPRL with the highly pure human PRL preparation (Figure 1c). All the control experiments were performed at least twice with 6 sections in each batch.

Of the 8 breast carcinoma cases positive reaction was seen in individual sections examined from 6 cases. Two of the positive cases had, in addition, strongly PRL positive apocrine change in the background breast tissue. The immunostained cells were found generally intermixed with variable numbers of unstained cancer cells. However, normal epithelial cells even within the tumorous areas were consistently negative. The specific staining was again mainly of the cytoplasmic type although in 2 specimens there was some irregular staining of the cell periphery as well as of some nuclei.

### Discussion

The results of this study indicate a fairly consistent presence of immunohistochemically demonstratable PRL in histologically definable apocrine metaplastic cells, particularly the papillary apocrine change variety, found within benign breast disease. The PRL immunostaining was also found with a high regularity in breast cancer specimens.

**Figure 1** Immunohistochemical demonstration of prolactin in apocrine metaplastic epithelium: (a) immunoperoxidase staining obtained using DNP-labelled rabbit anti-PRL serum (1:60,000, 16 h, 4°C) applied in conjunction with the modified DHSS procedure. Note the restriction of the staining to the exuberantly metaplastic cyst-lining cells; (b) marked and selective enhancement of the staining resulting from prior incubation of a serial adjacent section with highly pure human prolactin ($10\,\mu g\,mg^{-1}$, 1 h, 20°C); (c) abolition of the staining by preabsorption of the antiserum with the highly pure human prolactin ($10\,\mu g\,ml^{-1}$, 30 min, 37°C).

We have employed a highly sensitive immunohistochemical method for the examination of PRL immuno reactivity in formalin fixed and paraffin wax embedded breast sections. Our success with formalin fixed paraffin wax embedded tissue is, however, contrary to a previous report where the use of fresh frozen tissue was found essential (Dhadly & Walker, 1983). Nevertheless, Purnell *et al.* (1982) were able to demonstrate cytoplasmic PRL binding in paraffin wax embedded tissue but using an antiserum against bovine PRL. Similar results have been also reported by Marchetti *et al.* (1984) using both fresh and formalin fixed tissue. Staining of the epithelial cells of ducts and lobules seen in normal and hyperplastic benign breast tissue was described in these studies. These authors have not, however, specifically commented on any staining reaction associated with apocrine epithelium (i.e. if present) in the tissues examined by them. On the other hand, in our study the normal acinar or ductular epithelium did not stain significantly with aPRL.

The immunohistochemically detectable presence of PRL within the apocrine metaplastic cells a priori may imply either a target status, transcellular transport mechanism, de novo synthesis or degradation function for these cells with regard to this hormone. The fact that addition of exogenous PRL onto the sections led to considerable selective enhancement of the anti-PRL mediated staining of the apocrine metaplastic cells indicates that the cells are involved in the production of PRL specific binding sites, probably PRL receptors. If so, this could suggest a target function on the part of metaplastic cells for PRL. In situ hybridisation studies with a gene probe directed against the messenger RNA of PRL receptor may be one way of directly confirming this possibility on the available amounts of tissue material.

The breast is under cyclical control of various hormones throughout the reproductive life of a woman, yet consistent changes in basal plasma hormone levels have not been detected in breast disease. Recently women with cyclical mastalgia and nodularity have been shown to exhibit an increased PRL response to TRH or domperidone provocation (Kumar *et al.*, 1984). Prolactin is known to exert several physiological actions on breast cells (Shiu &

Friesen, 1980). PRL regulates water and electrolyte balance, milk protein synthesis, uridine conversion and incorporation into DNA, and breast fatty acid synthetase activity. An increased synthesis of oestrogen receptor has also been reported. The role of PRL in the overall causation of benign breast pathology is uncertain. However, treatment with bromocriptine, a PRL lowering dopaminergic agent was successful in alleviating painful nodularity and cyclical mastalgia (Mansel *et al.*, 1978). The determination and the level of specific PRL binding sites in normal and abnormal breast tissues may therefore be useful in better understanding of the patho-physiology of benign breast disease. The weak and rather heterogenously distributed PRL staining in the other types of BBD lesions, e.g. epitheliosis and sclerosing adenosis, is difficult to rationalise because of the very few instances in which it was observed.

The significance of PRL presence in apocrine metaplastic cells of BBD in relation to the possible association of apocrine metaplasia with breast cancer development is discussed briefly below.

Apocrine metaplasia or pink cell change, characterized by high cylindrical cells with granular eosinophlic cytoplasm projecting as 'snouts' into the lumen of the ducts and cysts, has long been regarded of no significance in cancer risk. Similarly breast carcinoma originating from apocrine metaplasia has long been considered to be a relatively rare event (Foote & Stewart, 1945). However, Haagensen has recently reported a very much higher incidence of apocrine type of breast carcinoma. For example, 60% apocrine differentiation of cancer cells was seen in a series where cancer developed in patients with concomitant gross cystic disease and 26% carcinomas with apocrine features were seen in an unselected series of 124 cases (Haagensen, 1986). Increased cancer risk of 2 to 10 times has been reported recently in 2 other independent studies involving long term follow-ups of patients whose initial biopsy had shown apocrine metaplasia (Page *et al.*, 1978; Roberts *et al.*, 1984). However, Dupont & Page (1985) in an updated study have regarded apocrine metaplasia as a non-proliferative benign breast lesion without a significant risk for breast cancer. Nevertheless, the frequency of apocrine differentiation in breast carcinomas assessed on purely morphological grounds is controversial (Craigmyle, 1984). The need to have more objective criteria of defining apocrine cell types under the light microscope has been emphasised in a recent article (Eusebi *et al.*, 1984). The availability of an antibody specific for apocrine carcinoma reported in the latter article is likely to help to determine the true level of association between apocrine metaplasia and breast cancer in general.

The high proportion of the breast cancer tissue staining for PRL observed in this study is in keeping with the findings of previous workers (Dhadly & Walker, 1983; Marchetti *et al.*, 1984; Purnell *et al.*, 1982). Also it is possible that the PRL positive breast cancer cells seen in the present study may represent cells depicting apocrine type breast carcinoma differentiation. This possibility needs to be examined by independent markers such as the antibody to GCDFP-15, a specific marker for apocrine metaplasia described by Haagensen and co-workers (Haagensen, 1986; Eusebi *et al.*, 1984). However, it is of interest to note that at least 2 out of 6 carcinoma cases positive for PRL staining were recognised to have PRL positive apocrine change in the background breast tissue despite being sampled on a limited and a rather random basis. A systematic approach to estimating the true incidence of PRL positive apocrine change in the background breast tissue of breast cancer patients is clearly called for in the future.

### References

CRAIGMYLE, M.B. (1984). *The apocrine glands and the breast.* Chichester: John Wiley & Sons p. 72.

DHADLY, M.S. & WALKER, R.A. (1983). The localisation of prolactin binding sites in human breast tissue. *Int. J. Cancer*, **31**, 433.

DIXON, J.M., LUMSDEN, A.B. & MILLER, W.R. (1985) The relationship of cyst type to risk factor for breast cancer and the subsequent development of breast cancer in patients with breast cystic disease. *Euro. J. Cancer Clin. Oncol.*, **21**, 1047.

DUPONT, W.D. & PAGE, D.L. (1985). Risk factors for breast cancer in women with proliferative breast disease. *N. Engl. J. Med.*, **312**, 146.

EUSEBI, V., BETTS, C., HAAGENSEN, D.E., Jr., GUGLIOTTA, P., BUSSOLATI, G., & AZZOPARDI, J.G. (1984). Apocrine differentiation in lobular carcinoma of the breast: a morphological, immunological and ultrastructural study. *Human Pathol.*, **15**, 134.

FOOTE, F.W. & STEWART, F.W. (1945). Comparative studies of cancerous versus non-cancerous breasts. I. Basic morphologic characteristics. *Ann. Surg.*, **121**, 6.

HAAGENSEN, C.D. (1986). *Diseases of the breast.* p. 95. W.B. Saunders Co., Philadelphia.

HEWLINS, M.J.E., WEEKS, I. & JASANI, B. (1984). Non-deleterious dinitrophenyl (DNP) hapten labelling of antibody protein. Preparation and properties of some short-chain DNP imidoesters. *J. Immunol. Meth.*, **70**, 111.

JASANI, B., THOMAS, D.W. & WILLIAMS, E.D. (1981). Use of monoclonal antihapten antibodies for immunolocalisation of tissue antigens. *J. Clin. Pathol.*, **34**, 1000.

JASANI, B. & WILLIAMS, E.D. (1985). Hapten enzyme labelling. British Patent No. 2098, 730B.

JASANI, B., EDWARDS, R.E., THOMAS, N.D. & GIBBS, A.R. (1985). The use of vimentin antibodies in the diagnosis of malignant mesothelioma. *Virchows Arch.* (*Pathol. Anat.*), **406**, 441.

KUMAR, S., MANSEL, R.E., HUGHES, L.E., WOODHEAD, J.S., EDWARDS, C.A., SCANLON, M.F. & NEWCOMBE, R.G. (1984). Prolactin response to thyrotropin-releasing hormone stimulation and dopaminergic inhibition in benign breast disease. *Cancer*, **53**, 1311.

MANSEL, R.E., PREECE, P.E. & HUGHES, L.E. (1978). A double blind trial of the prolactin inhibitor bromocriptine in painful benign breast disease. *Br. J. Surg.*, **65**, 724.

MARCHETTI, E., RIMONDI, A.P., QUERZOLI, P., FABRIS, G. & NENCI, I. (1984). Prolactin and prolactin binding sites in human breast cancer cells. In *Progress in clinical and biological research. Hormones and Cancer*. Curpride *et al.* (eds), **142**, 109. Allan R. Liss Inc., New York.

PAGE, D.L. & DUPONT, W.D. (1986). Are breast cysts a premalignant marker? *Eur. J. Cancer Clin. Oncol.*, **22**, 635.

PAGE, D.L., ZWAGG, R.V., ROGERS, L.W., WILLIAMS, L.T., WALKER, W.E. & HARTMAN, W.H. (1978). Relation between component parts of fibrocystic disease complex and breast cancer. *J. Natl Cancer Inst.*, **61**, 1055.

PURNELL, D.M., HILLMAN, E.A., HEATFIELD, B.M. & TRUMP, B.F. (1982). Immunoreactive prolactin in epithelial cells of normal and cancerous human breast and prostate detected by the unlabelled antibody peroxidase–antiperoxidase method. *Cancer Res.*, **42**, 2317.

ROBERTS, M.M., JONES, V., ELTON, R.A., FORTT, R.W., WILLIAMS, S. & GRAVELLE, I.H. (1984). Risk of breast cancer in women with history of benign disease of the breast. *Br. Med. J.*, **288**, 275.

SHIU, R.P.C. & FRIESEN, H.G. (1980). Mechanism of action of prolactin in the control of mammary gland function. *Ann. Rev. Physiol.*, **42**, 83.


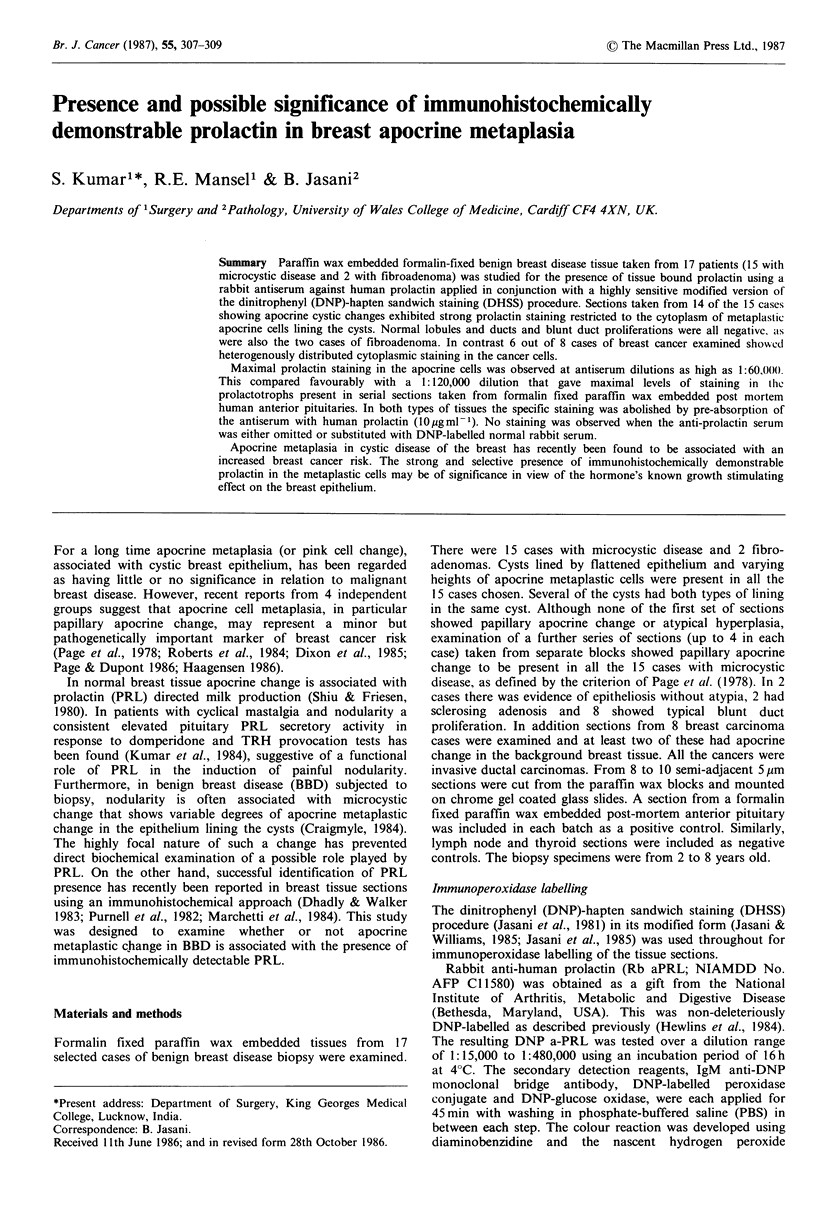

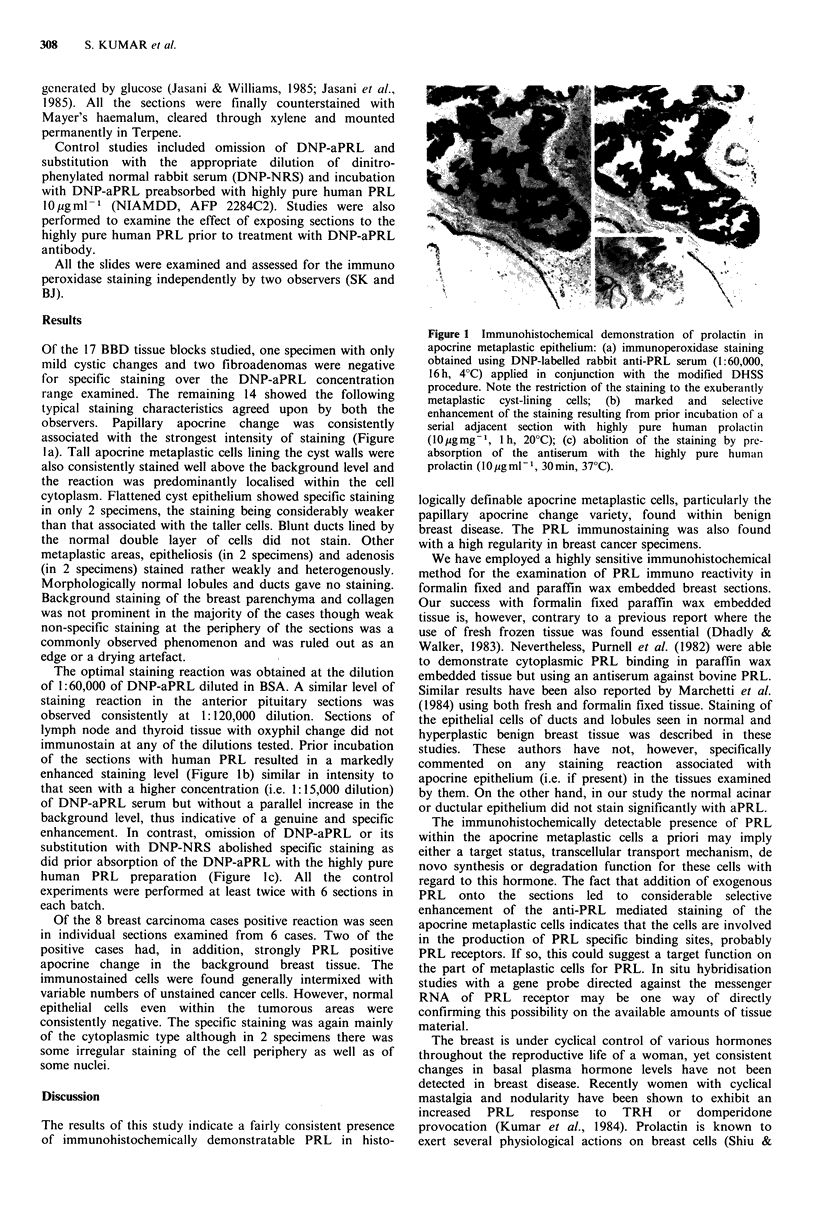

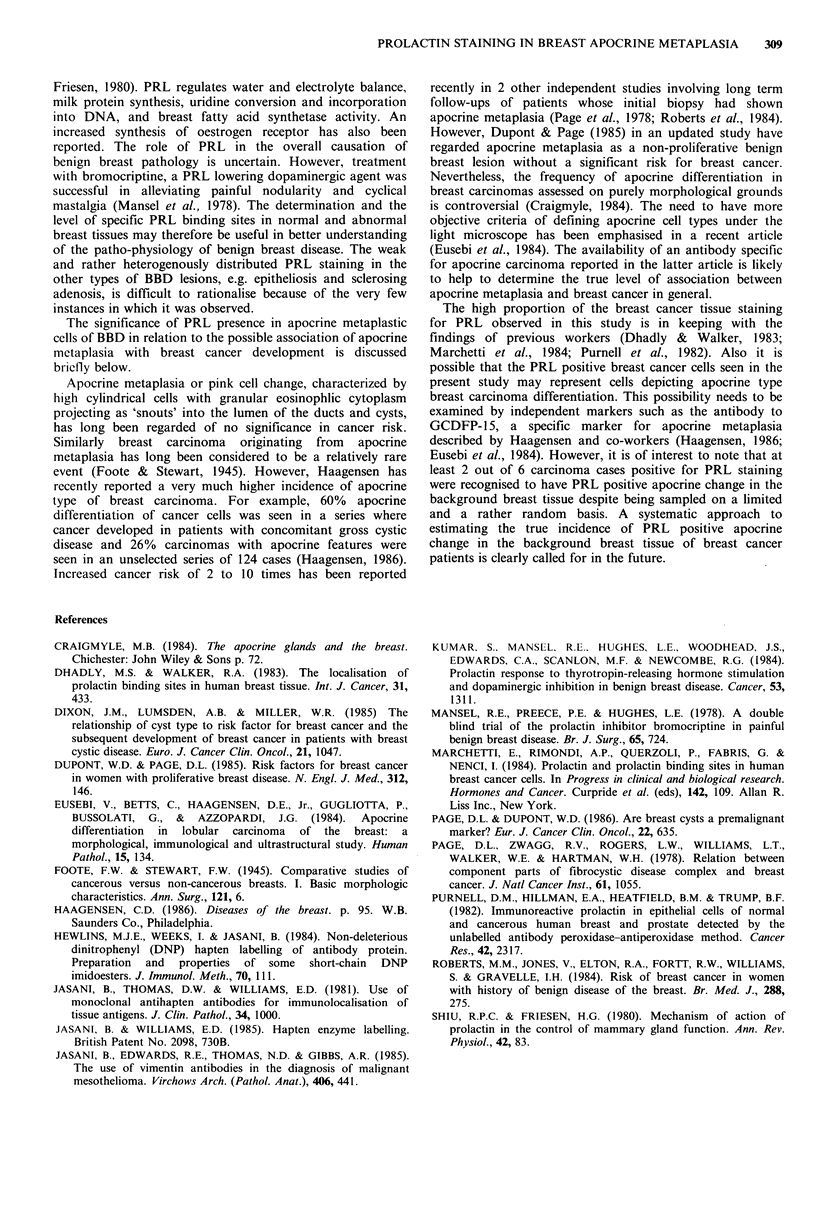


## References

[OCR_00344] Dhadly M. S., Walker R. A. (1983). The localization of prolactin binding sites in human breast tissue.. Int J Cancer.

[OCR_00349] Dixon J. M., Lumsden A. B., Miller W. R. (1985). The relationship of cyst type to risk factors for breast cancer and the subsequent development of breast cancer in patients with breast cystic disease.. Eur J Cancer Clin Oncol.

[OCR_00355] Dupont W. D., Page D. L. (1985). Risk factors for breast cancer in women with proliferative breast disease.. N Engl J Med.

[OCR_00360] Eusebi V., Betts C., Haagensen D. E., Gugliotta P., Bussolati G., Azzopardi J. G. (1984). Apocrine differentiation in lobular carcinoma of the breast: a morphologic, immunologic, and ultrastructural study.. Hum Pathol.

[OCR_00367] Foote F. W., Stewart F. W. (1945). Comparative Studies of Cancerous Versus Noncancerous Breasts.. Ann Surg.

[OCR_00376] Hewlins M. J., Weeks I., Jasani B. (1984). Non-deleterious dinitrophenyl (DNP) hapten labelling of antibody protein. Preparation and properties of some short-chain DNP imidoesters.. J Immunol Methods.

[OCR_00391] Jasani B., Edwards R. E., Thomas N. D., Gibbs A. R. (1985). The use of vimentin antibodies in the diagnosis of malignant mesothelioma.. Virchows Arch A Pathol Anat Histopathol.

[OCR_00382] Jasani B., Thomas D. W., Williams E. D. (1981). Use of monoclonal antihapten antibodies for immunolocalisation of tissue antigens.. J Clin Pathol.

[OCR_00398] Kumar S., Mansel R. E., Hughes L. E., Woodhead J. S., Edwards C. A., Scanlon M. F., Newcombe R. G. (1984). Prolactin response to thyrotropin-releasing hormone stimulation and dopaminergic inhibition in benign breast disease.. Cancer.

[OCR_00403] Mansel R. E., Preece P. E., Hughes L. E. (1978). A double blind trial of the prolactin inhibitor bromocriptine in painful benign breast disease.. Br J Surg.

[OCR_00408] Marchetti E., Rimondi A. P., Querzoli P., Fabris G., Nenci I. (1984). Prolactin and prolactin binding sites in human breast cancer cells.. Prog Clin Biol Res.

[OCR_00415] Page D. L., Dupont W. D. (1986). Are breast cysts a premalignant marker?. Eur J Cancer Clin Oncol.

[OCR_00419] Page D. L., Vander Zwaag R., Rogers L. W., Williams L. T., Walker W. E., Hartmann W. H. (1978). Relation between component parts of fibrocystic disease complex and breast cancer.. J Natl Cancer Inst.

[OCR_00425] Purnell D. M., Hillman E. A., Heatfield B. M., Trump B. F. (1982). Immunoreactive prolactin in epithelial cells of normal and cancerous human breast and prostate detected by the unlabeled antibody peroxidase-antiperoxidase method.. Cancer Res.

[OCR_00432] Roberts M. M., Jones V., Elton R. A., Fortt R. W., Williams S., Gravelle I. H. (1984). Risk of breast cancer in women with history of benign disease of the breast.. Br Med J (Clin Res Ed).

[OCR_00438] Shiu R. P., Friesen H. G. (1980). Mechanism of action of prolactin in the control of mammary gland function.. Annu Rev Physiol.

